# A Combination of *Ex vivo* Diffusion MRI and Multiphoton to Study Microglia/Monocytes Alterations after Spinal Cord Injury

**DOI:** 10.3389/fnagi.2017.00230

**Published:** 2017-07-19

**Authors:** Harun N. Noristani, Hassan Boukhaddaoui, Guillaume Saint-Martin, Pauline Auzer, Rahima Sidiboulenouar, Nicolas Lonjon, Eric Alibert, Nicolas Tricaud, Christophe Goze-Bac, Christophe Coillot, Florence E. Perrin

**Affiliations:** ^1^Institut National de la Santé et de la Recherche Médicale, U1051 Montpellier, France; ^2^University of Montpellier, Montpellier; Institut National de la Santé et de la Recherche Médicale, U1198, Montpellier; École Pratique des Hautes Études Paris, France; ^3^Charles Coulomb Laboratory, UMR 5221 Centre National de la Recherche Scientifique Montpellier, France; ^4^Centre Hospitalier Universitaire de Montpellier (CHRU), Gui de Chauliac Hospital Montpellier, France

**Keywords:** spinal cord injury, microglia/monocytes, *ex vivo* diffusion MRI, tissue clearing, two-photon

## Abstract

Central nervous system (CNS) injury has been observed to lead to microglia activation and monocytes infiltration at the lesion site. *Ex vivo* diffusion magnetic resonance imaging (diffusion MRI or DWI) allows detailed examination of CNS tissues, and recent advances in clearing procedures allow detailed imaging of fluorescent-labeled cells at high resolution. No study has yet combined *ex vivo* diffusion MRI and clearing procedures to establish a possible link between microglia/monocytes response and diffusion coefficient in the context of spinal cord injury (SCI). We carried out *ex vivo* MRI of the spinal cord at different time-points after spinal cord transection followed by tetrahydrofuran based clearing and examined the density and morphology of microglia/monocytes using two-photon microscopy. Quantitative analysis revealed an early marked increase in microglial/monocytes density that is associated with an increase in the extension of the lesion measured using diffusion MRI. Morphological examination of microglia/monocytes somata at the lesion site revealed a significant increase in their surface area and volume as early as 72 hours post-injury. Time-course analysis showed differential microglial/monocytes response rostral and caudal to the lesion site. Microglia/monocytes showed a decrease in reactivity over time caudal to the lesion site, but an increase was observed rostrally. Direct comparison of microglia**/**monocytes morphology, obtained through multiphoton, and the longitudinal apparent diffusion coefficient (ADC), measured with diffusion MRI, highlighted that axonal integrity does not correlate with the density of microglia/monocytes or their somata morphology. We emphasize that differential microglial/monocytes reactivity rostral and caudal to the lesion site may thus coincide, at least partially, with reported temporal differences in debris clearance. Our study demonstrates that the combination of *ex vivo* diffusion MRI and two-photon microscopy may be used to follow structural tissue alteration. Lesion extension coincides with microglia/monocytes density; however, a direct relationship between ADC and microglia/monocytes density and morphology was not observed. We highlighted a differential rostro-caudal microglia/monocytes reactivity that may correspond to a temporal difference in debris clearance and axonal integrity. Thus, potential therapeutic strategies targeting microglia/monocytes after SCI may need to be adjusted not only with the time after injury but also relative to the location to the lesion site.

## Introduction

Spinal cord injury (SCI) is a devastating neuropathology affecting over 2.5 million people worldwide (van den Berg et al., 2010). Depending on the anatomical level and the severity of the spinal cord lesion, clinical symptoms range from minor sensory/motor damage to complete quadriplegia.

In clinic, magnetic resonance imaging (MRI) is the only non-invasive method for examining the impact of the insult on the structure and function of human spinal cord (Wheeler-Kingshott et al., 2014). MRI is also well adapted for following the evolution of the lesion in pre-clinical animal models of SCI (Bonny et al., 2004; Denic et al., 2011). In particular, *ex vivo* MRI analysis provides high resolution images of the spinal cord, allowing accurate assessment of lesion-induced tissue alterations. Recently, we have shown that *ex vivo* ^1^H-MRI (9.4T) closely correlates with histological examination of the injured spinal cord after different lesion severities (Noristani et al., 2015).

Diffusion weighted MRI (diffusion MRI, or DWI) reflects the local dynamics of water molecules in the tissue. The structure of tissues can be characterized by the capability of water to diffuse along preferential directions and can be quantitatively assessed using the apparent diffusion coefficient (ADC). DWI thus offers information on tissue integrity and structure that cannot be obtained by conventional ^1^H-MRI. In particular, the longitudinal ADC (lADC), which represents the diffusivity along the rostro-caudal axis and reflects the integrity of white matter fibers, is often decreased in the presence of axonal injury (Song et al., 2003; Vedantam et al., 2014). In the spinal cord, diffusion MRI is often applied to evaluate myelin integrity (Bonny et al., 2004) but may also be applied to assess morphological alterations of cell populations, including glia. In addition, diffusion MRI has been used to examine the 3-dimensional (3D) structure and architecture of post-mortem brains for review, [see Bastiani and Roebroeck (2015)].

Numerous animal models have been developed to study SCI pathophysiology including spinal cord compression, contusion, ischemia and transection (Lee and Lee, 2013). Whilst these animal models partially recapitulate clinical symptoms, they have important limitations due to the high level of inter-individual differences in the severity of the lesion (Basso et al., 1996; Onifer et al., 2007). Although transection of the spinal cord is regarded as the most severe model of SCI, it has the advantage of minimizing the inter-individual differences in injury severity (M'Dahoma et al., 2014) and is more easily quantified using MRI (Wang et al., 2014). Because of its high reproducibility, transection of the spinal cord reduces the need for a large number of animals to obtain biologically robust results. The majority of studies using transection as SCI models have focused on rats (Basso et al., 1996; Antri et al., 2005; Onifer et al., 2007; M'Dahoma et al., 2014); however, transgenic mice offer advantages to better understand the pathophysiological mechanisms involved in SCI.

SCI pathophysiology includes damage to the blood-spinal cord barrier, monocytes infiltration at the lesion site and elevated immune response, all contributing to neuronal demise. In addition, SCI triggers the pronounced activation of glial cells, including microglia. Microglia, the resident immune cells of the central nervous system (CNS) are the first activated glial population observed after SCI (Tian et al., 2007) and contribute to monocytes-derived macrophages recruitment to the lesion site. Under physiological conditions, microglia display a ramified morphology with small somata and long/thin cellular processes that continuously scan their territorial domains (Kettenmann et al., 2011). Following SCI, microglia migrate toward the lesion site, where they acquire an amoeboid morphology with large somata and short/thick processes (Tian et al., 2007; Noristani et al., 2017). One means of studying microglia is to use CX3CR1^eGFP^ transgenic mice, which express enhanced green fluorescent protein (eGFP) downstream of the Cx3cr1 promoter, a chemokine receptor highly expressed in microglia, circulating monocytes and resident macrophages (Jung et al., 2000; Gautier et al., 2012; Wolf et al., 2013). Microglia and peripheral monocytes of common myelomonocytic origin display similar morphologies and express common surface receptors and signaling molecules (Schmitz et al., 2009; Kettenmann et al., 2011). Recent reports identified potential markers, including Ly6C, to distinguish between microglia and monocytes (Chiu et al., 2013; Butovsky et al., 2014; Gosselin et al., 2014; Bennett et al., 2016); however, to our knowledge, no transgenic mice expressing a fluorescent marker downstream of Ly6C have been generated.

Although the activation of microglia/monocytes after SCI have been acknowledged for almost a century, little is currently known about their density after injury and their longitudinal repartition along the spinal cord axis rostral and caudal to the lesion site (Schnell et al., 1999; Kigerl et al., 2006; Stirling and Yong, 2008). Earlier studies using semi-quantitative histology reported a time-dependent increase in microglia/monocytes density up to 7 days after dorsal spinal cord hemisection in mice (Stirling and Yong, 2008). Other histological study revealed a peak of microglia/monocyte reactivity in mice between 7 and 14 days post-injury after contusion of the spinal cord (Kigerl et al., 2006). More recent studies using quantitative flow cytometry have shown that the increased microglia/monocytes density observed within 2 days post-contusion injury in mice is subsequently reduced starting from 4 days post-SCI (Stirling and Yong, 2008). Contradictory findings have also been reported in rats after contusion SCI with a peak in microglia/monocyte density between 2 (Abdanipour et al., 2013) and 7 days post-injury (Beck et al., 2010).

To develop efficient therapies that target the inflammatory response after SCI, it is necessary to acquire precise knowledge of microglia/monocytes density, not only at acute and chronic stages post-injury but also at different distances from the lesion epicenter. Previous studies on microglia/monocyte density after SCI relied on histology combined with manual cell counting (Schnell et al., 1999; Kigerl et al., 2009; Adamczak et al., 2010), which provides only semi-quantitative data, at best. In addition, standard histological analyses require tissue sectioning, making 3D analysis of microglia/monocytes throughout the whole tissue virtually impossible. Clearing, a recent advancement in tissue processing that renders intact organs transparent, allows fast 3D analyses of the entire spinal cord (Erturk et al., 2012; Chung et al., 2013; Renier et al., 2014; Tomer et al., 2014; Yang et al., 2014).

To date, only one study has reported changes in microglia/monocytes density after spinal cord hemisection in mice using the clearing approach (Erturk et al., 2012). However, the authors analyzed only a single time-point (10 days after lesion) with no indications of microglia/monocytes density at either acute or more chronic stages post-injury. In addition, Erturk et al. (2012) mainly focused on the lesion epicenter and did not provide data regarding microglia/monocytes density at more distal locations (Erturk et al., 2012). Given the dynamic reactivity of microglia/monocytes, it is necessary to study their alterations in acute and chronic stages after SCI and at different distances both rostral and caudal to the lesion site. Finally, even if numerous studies have reported microglia/monocytes activation after SCI, none has quantified their morphological changes after injury.

Our aim was to examine detailed SCI-induced tissues and cellular alterations at different time-points and distances from the lesion site using (a) high resolution *ex vivo* diffusion MRI and (b) two-photon imaging of cleared spinal cords to evaluate 3D changes in microglia/monocytes density and morphology. Our specific objective was to identify a possible correlation between *ex vivo* diffusion MRI signals, which reflect the structure of tissues, and microglial/monocytes density/morphology. Since microglia are involved in debris clearance, we hypothesized that lesion size may be related, at least partially, to microglia reactivity. We thus first compared modifications in microglia/monocytes density and morphology to the extension of the lesion evaluated by DWI. We then compared the density and the morphology of microglia/monocytes at different distances on both sides of the lesion. Finally, we tried to determine whether or not axonal integrity, reflected by the lADC, correlates with microglia density and morphology. We found that microglial/monocytes density coincides with lesion extension; however, lADC does not seems to correlate with microglia/monocyte density and morphology. We also established that microglial/monocytes somata morphology is different rostral and caudal to the lesion epicenter. Our results constitute the first detailed 3D analysis of a cell populations in the spinal cord after lesion using a combination of *ex vivo* diffusion MRI and two-photon microscopy.

## Materials and methods

### Ethic approval

Experimental procedures followed the European legislative, administrative and statutory measures for animal experimentation (86/609/EEC). The study was approved by the “Direction des Services Vétérinaires de l'Hérault” and the “Ministère de l'Education Nationale, de l'Enseignement Supérieur et de la Recherche, ethics committee N°36,” France (authorization number 34118).

### Animals and spinal cord injury

Transgenic mice expressing enhanced green fluorescent protein (eGFP) in microglia/monocytes (CX3CR1^+/eGFP^) (Jung et al., 2000) were obtained from Dr. Dan Littman, Howard Hughes Medical Institute, Skirball Institute, NYU Medical Centre, New York, USA and maintained on a C57BL/6 background (The Jackson Laboratory, Bar Harbor, ME, USA). Heterozygous eGFP^−/+^ mice were used in the current study. All mice were housed in controlled conditions (hygrometry, temperature and 12-h light/dark cycle) with free access to food and water. Adult male mice (12 weeks of age) were anesthetized by inhalation of 1.5% isoflurane (Aerane, Baxter, Deerfield, IL, USA). The skin and muscles overlying the low thoracic segment of the rachis were cut, followed by a dorsal laminectomy of the thoracic 9 (T9) vertebra. Meninges were incised and transection of the spinal cord was carried out under a microscope using a micro-scalpel (FST, Heidelberg, Germany). Lesions were made at T9 level to obtain complete paraplegia whilst preserving full respiratory function. Following lesion, muscles and skin were sutured and animals were left to recover on a heated surface (not exceeding 38°C). Bladders were emptied manually twice daily and bodyweight was measured throughout the study period. Three time-points were chosen to reveal microglia/monocytes changes both at acute (72 hours) and chronic (4 and 6 weeks) stages after severe spinal cord lesion. Non-injured group served as control (*n* = 4 for each group).

### Tissue processing

Mice were deeply anesthetized via intraperitoneal injection of tribromoethanol (500 mg/kg) followed by transcardial perfusion using cold 0.1 M phosphate buffer saline (PBS) at pH 7.2 and 4% paraformaldehyde (PFA, Sigma Aldrich, Saint Louis, USA). The entire spinal cords were then dissected and post-fixed in the 4% PFA for an additional 2 h.

### *Ex vivo* diffusion weighted imaging

**Figure 1:**
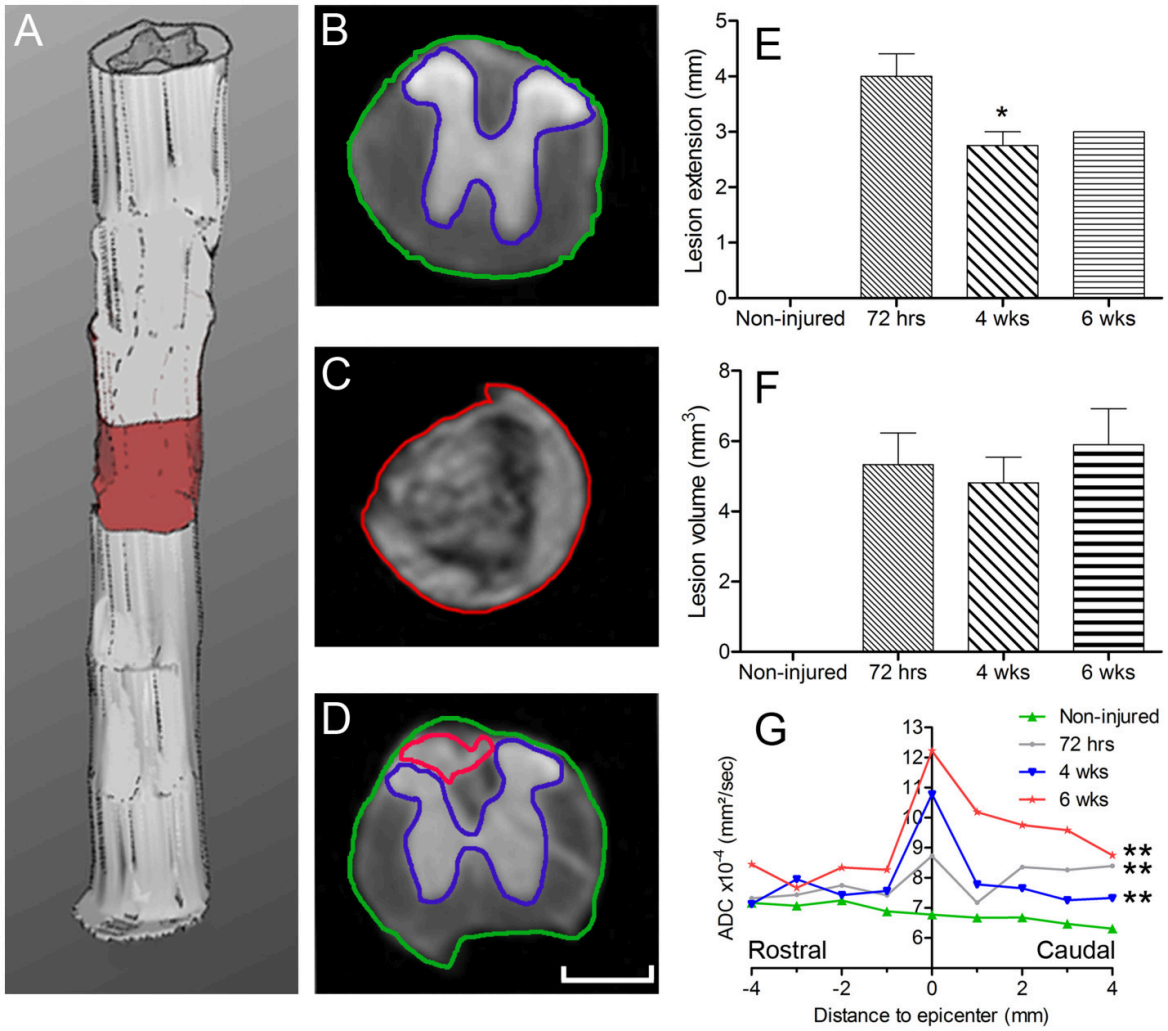
*Ex vivo* diffusion ^1^H-MRI assessments following SCI. 3D reconstruction of an injured spinal cord **(A)**, the red area represents lesion extension. Images of *ex vivo* diffusion MRI from mouse spinal cord rostral **(B)** within **(C)** and caudal **(D)** to the lesion epicenter. Note the excellent anatomical resolution of the spinal cord that clearly distinguishes the entire spinal cord (outlined in green in **B** and **D**), damaged spinal cord tissues (outlined in red in **C** and **D**) as well as the intact gray matter (outlined in blue in **B** and **D**). Bar graph displaying the evolution of the extension of the lesion at different stages after complete SCI **(E)**. Bar graph showing changes in the volume of the lesion at different time-points after injury **(F)**. Line graph displaying changes in apparent diffusion coefficients along the spinal cord at different stages after SCI **(G)**. Statistics: ^*^P < 0.05 and ^***^P < 0.001. **(E)**, 4 weeks compared to 72 hours post-lesion, un-paired *t*-test. **(G)**, All groups are compared to the non-injured control, Wilcoxon signed-rank test.

Spinal cords were positioned in a 5-mm-diameter glass tube filled with 0.1 M PBS surrounded by a custom-made ribbon solenoid coil (40 mm in length, 8 mm outer diameter and 4 turns, Supplementary Figure 1K) (Coillot et al., 2016) and imaged using 9.4 Tesla apparatus (Agilent Varian 9.4/160/ASR, Santa Clara, California, USA) associated with VnmrJ imaging acquisition system (Agilent, Palo Alto, California, USA). The use of the custom-made ribbon solenoid coil enhanced signal-to-noise ratio (SNR) and, drastically reduced acquisition time. Specifically, for this experiment, SNR was increased by 7 compared to a regular 43 mm quadrature volumic coil. We first acquired high resolution T2 weighed ^1^H-MRI using a spin echo sequence to obtain MR images without diffusion-weighting (diffusion gradient: G = 0 G.cm^−1^). The following acquisition parameters were used: delta = 6.88 ms, G = 0 G/cm^−1^, separation = 15.05 ms, (TR) repetition time = 1,580 ms, (TE) echo time = 30.55 ms, AVG = 30, FOV = 10 × 10 mm, slices = 36, thickness = 1 mm without gap and acquisition matrix = 128 × 128. Scanning time: approximately 90 min. Diffusion-weighted MRI sequence was then used to acquire DWI and to calculate the ADC. ADC is a quantitative measurement of local water diffusion (mm^2^.s^−1^) on a given region and direction which is sensitive to the local structure and anisotropy of tissues. ADC results from the combination of acquisitions with (S at *b* = 500) and without (S0 at *b* = 0) diffusion gradient. To acquire axial images, the sensitivity of diffusion MRI was preliminary evaluated in three directions including the longitudinal axis of the spinal cord. Longitudinal DWI offered better signal-to-noise ratio and image contrast to discriminate between the intact and the damaged regions of the spinal cord. We thus acquired DWI only on the longitudinal axis with the following parameters: magnetic field gradient G = 20 G.cm^−1^ during 6.88 ms, separation = 15.05 ms; (TR) repetition time = 1,580 ms, (TE) echo time = 30.55 ms, AVG = 30, FOV = 10 × 10 mm, slices = 36, thickness = 1 mm without gap and acquisition matrix = 128 × 128. Scanning time: approximately 180 min. Diffusion data were processed by using a MATLAB-based in-house toolbox. All MRI visualization and segmentation were done using Myrian software (Intrasense, Montpellier, France); intact (entire spinal cord, white and gray matters) and damaged tissues were manually surrounded (Figures 1B–D and Supplementary Figures 1A–J).

### Tissue clearing and two-photon image acquisition

**Figure 2:**
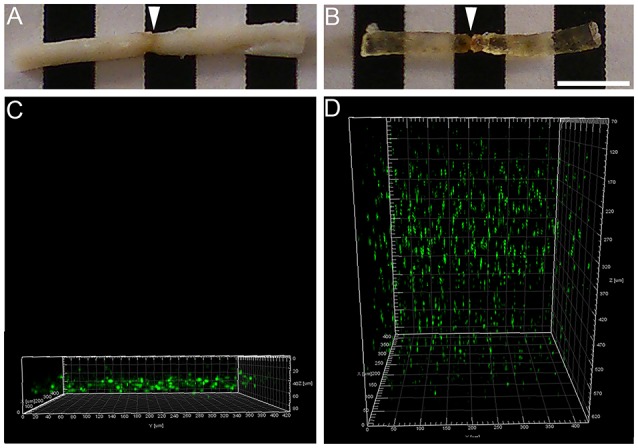
Two-photon imaging of the spinal cord before and after clearing. Photographs showing an injured spinal cord (arrows) before **(A)** and after **(B)** clearing. Using two-photon microscopy, the spinal cord was imaged before and after clearing. Two-photon micrographs displaying eGFP-expressing microglia/monocytes before **(C)** and after **(D)** clearing. Note that, before clearing, <100 μm of the spinal cord's thickness could be imaged, while clearing allowed imaging of the entire thickness of the spinal cord. X axis: perpendicular to the sample; Y axis: longitudinal to the sample and Z axis represent the depth of imaging. Scale bars **(A,B)**: 5 mm.

After diffusion MRI acquisition, spinal cords were first rinsed in 0.1 M PBS and clarified using the 3-dimensional imaging of solvent cleared organs (3DISCO) procedure, as previously described (Erturk et al., 2012). Briefly, spinal cords were incubated in 50, 70, and 80% tetrahydrofuran (THF, Sigma Aldrich, Saint Louis, USA) followed by three incubations in 100% THF (30 min each). Spinal cords were then placed in dichloromethane (Sigma Aldrich, Saint Louis, USA) for 30 min and finally in dibenzyl ether (DBE, Sigma Aldrich, Saint Louis, USA) for 20–30 min, until the samples became transparent (Figure 2). All incubation steps were done in the dark; spinal cords were placed in glass vials on a rotator. Samples were then mounted on a glass slide in DBE and coversliped, then immediately imaged using a two-photon scanning microscope (Zeiss LSM 7MP) equipped with a femtosecond pulsed Ti:Sapphire laser (Ultra II, Coherent) at 950 nm. We scanned at a 1,024 × 1,024 pixel resolution and 5 μm z steps; full 3D scan took 20–30 min.

### Microglial/monocytes quantification

**Figure 3:**
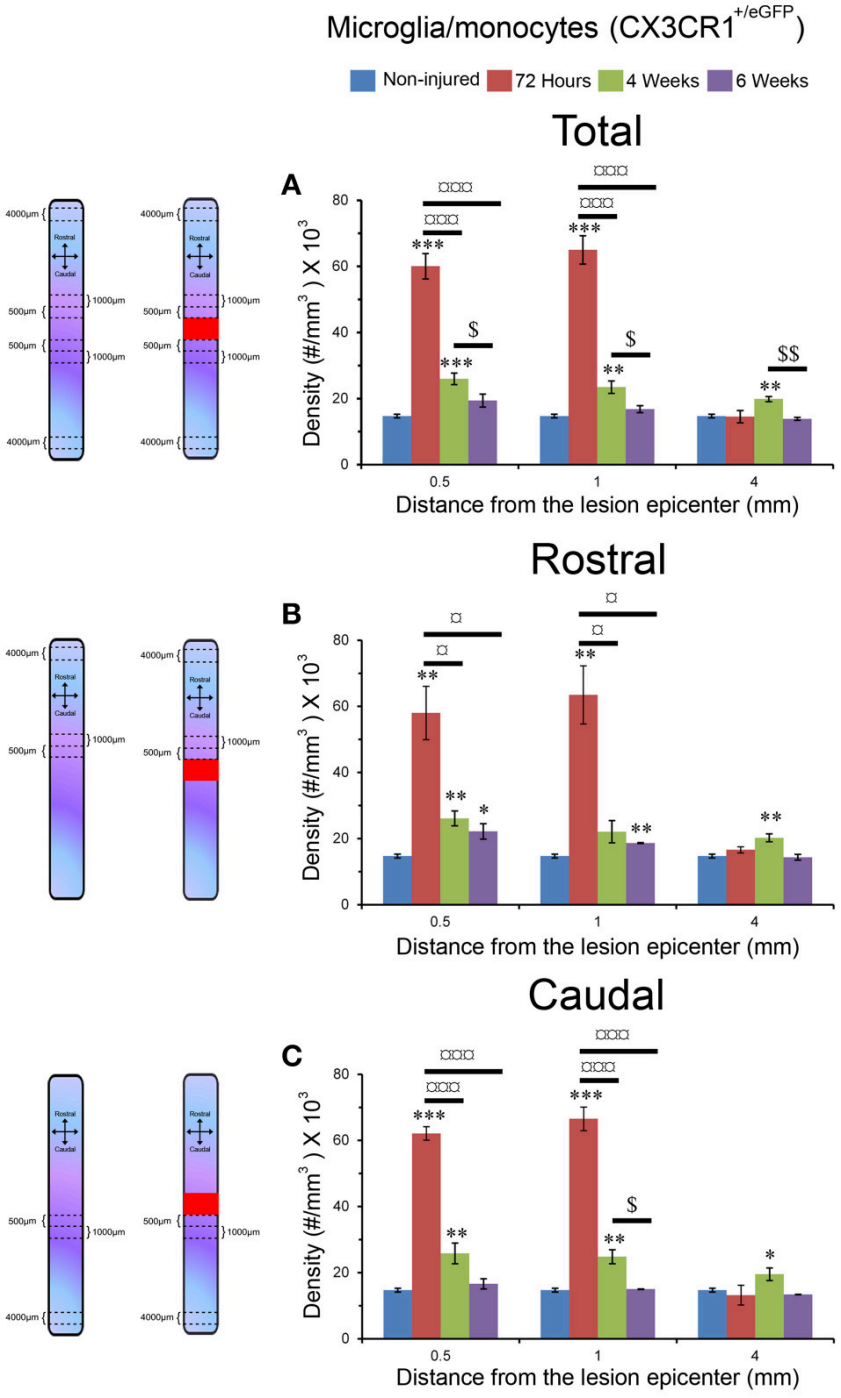
Increased microglia/monocytes density early after SCI. Schematic drawing of longitudinal spinal cord from non-injured control and after transection illustrating the lesion site (red rectangle) and the different zones (dashed lines) where microglia/monocytes densities were measured. Bar graphs displaying SCI-induced changes in microglia/monocytes density at different time-points and distances from the lesion epicenter. A strong increase in microglia/monocytes density is observed at 72 hours post-lesion both rostral and caudal to the lesion site **(A–C)**. ^*^P < 0.05, ^**^P < 0.01, ^***^P < 0.001 compared to un-injured control, ^¤^*P* < 0.05, ^¤¤¤^*P* < 0.001 compared to 72 h post-lesion, ^$^*P* < 0.05 compared to 4 weeks post-lesion group, un-paired *t*-test.

To quantify microglia/monocytes density (numerical density (N_v_), number (#)/mm^3^) throughout the entire thickness of spinal cord, we chose 3 different zones located at different distances from the edge of the lesion site: 0–500 μm immediately adjacent to the injury site as well as 500-1,000 and 4,000–4,500 μm away from the lesion epicenter, both rostral and caudal to the injury site (Figure 3). For non-injured controls, we analyzed equivalent anatomical levels of the spinal cord. Tissue volumes and microglial/monocytes density were calculated using the 3D image processing software Imaris x64 7.2.2. (Bitplane AG, Zürich, Switzerland). Microglia/monocytes were converted into 3D objects for fully automated counting (Supplementary Video 1). To ensure accuracy of the counts, selected segments were rotated to different angles and assessed visually. Only a sub-population of microglia/monocytes with no overlapping processes were selected for morphological analysis of their somata. Microglial/monocytes somata morphology was measured in 190–300 cells per time-point and compared to those of the un-injured control. The original scan was also converted into 3D objects for automated morphological analysis. Given that microglial/monocyte activation is characterized by pronounced enlargement of their somata, morphological analysis was carried out using Imaris to determine somata surface area (μm^2^) and volume (μm^3^), as previously described (Rodriguez et al., 2015).

### Statistical analysis

Un-paired *t*-tests were used to compare differences between 2 groups. The Wilcoxon signed-rank test was used to compare ADC values between groups along an 8 mm segment centered on the lesion site. A paired-*t*-test was used to compare ADC values between groups in the rostral and caudal segments. Significance was defined as *p* ≤ 0.05.

All data were analyzed using GraphPad Prism 4.0 (GraphPad Software, Inc., CA, USA). Data are shown as the mean ± standard error of the mean (SEM).

## Results

### *Ex vivo* diffusion MRI allows quantification of lesion extension and volume

We acquired 36 axial diffusion MR images (1 mm thick) over a 3.6 cm segment centered on the lesion epicenter (Figure 1A). We then quantified the rostro-caudal extension of the lesion. Lesion segmentation was carried out manually by outlining the spared (Figures 1B,D, *green: total; blue: gray matter*) and injured (Figures 1C,D, *red*) tissues on axial images. The mean extension of the lesion at 72 hours was 4 ± 0.4 mm centered on the epicenter and decreased to 2.75 ± 0.25 and 3 ± 0 mm by 4 and 6 weeks, respectively, thus revealing a significant decrease in rostro-caudal extension of the lesion between 72 hours and 4 weeks post-injury, followed by a stabilization (Figure 1E). The mean lesion volume amounted to 5.3 ± 0.9, 4.8 ± 0.7, and 5.9 ± 1 mm^3^ at 72 hours, 4 and 6 weeks post-injury, respectively, and quantitative assessment showed no significant changes over time (Figure 1F).

### Water diffusion modification is time-dependent

We then quantified the diffusion of the water in the tissues using diffusion MRI and evaluated changes in ADC values. At the lesion site, no clear distinction between the gray and white matter was possible due to complete tissue damage (Figure 1C). ADC was measured on an 8 mm segment centered on the lesion site. Overall, non-injured spinal cords showed an ADC of 6.78 × 10^−4^ mm^2^/s (Figure 1G). The mean ADC value at the lesion epicenter reached 8.73, 10.78, and 12.22 × 10^−4^ mm^2^/s at 72 hours, 4 and 6 weeks, respectively (Figures 1C,G). Moreover, an overall increase in the ADC contiguous to the lesion site, both rostral and caudal, was observed from 72 hours up to 6 weeks post-injury (Figure 1G). For all post-injury time points, the ADC was higher in the caudal than in the rostral segments 4 mm away from the lesion site. Moreover, there was an increased ADC 6 weeks post-injury in the rostral segment, whereas the ADC in the caudal segment remained relatively stable after injury (Figure 1G). All injured groups displayed a significant increase in ADC compared to non-injured animals; however, no significant differences between post-injury time points were observed (Figure 1G, Table 1). A Wilcoxon signed-rank test showed a significant increase in ADC between 72 hours and 6 weeks post-injury (Figure 1G, Table 1) compared to non-injured animals. To provide a more in-depth analysis, we analyzed separately the rostral and caudal segments. A paired *t*-test revealed a significant increase in ADC 72 hours and 6 weeks post-injury in the caudal segment when compared to non-injured animals; conversely, the injury did not modify the ADC in the rostral segment (Table 1).

**Table 1:** Quantification of longitudinal ADC over time following SCI.

	**72 hours vs. NI**	**4 weeks vs. NI**	**6 weeks vs. NI**	**72 hours vs. 4 weeks**	**72 hours vs. 6 weeks**	**4 vs. 6 weeks**
Total: −4 to 4 mm (Wilcoxon)	^**^	^**^	^**^	ns	^**^	^**^
Rostral: −4 to 0 (paired *t*-test)	ns	ns	ns	ns	ns	ns
Caudal: 0 to +4 mm (paired *t*-test)	^**^	ns	^**^	ns	^*^	^***^

*Comparison between injured animals at different time-points post-injury and non-injured animals. The Wilcoxon signed-rank test was used to compare ADC values between groups along an 8 mm segment centered on the lesion site. “Total” represents the entire segment of the spinal cord (Figure 1G; −4 to +4 mm). A paired-t-test was used to compare ADC values between groups in the rostral and caudal segments. “Rostral” and “caudal” refer to quantifications on each side of the lesion (Figure 1G; rostral: −4 to lesion; caudal: +4 mm to lesion). Percentages represent the comparison with the value from un-injured control group. NI, non-injured, ns, non-significant*.

* P < 0.05 and

** *P < 0.01, un-paired t-test*.

Together, these data show that, in contrast to the quantification of the extension and the volume of the lesion, the quantification of water diffusion allows identification of time-dependent modifications. Moreover, we highlight differences in water diffusion between segments located symmetrically rostral and caudal to the lesion site.

### Increased microglia/monocytes density after complete SCI

We used tetrahydrofuran (THF)-based clearing to render the adult spinal cord transparent while preserving eGFP signal in microglia/monocytes (Figure 2). While un-cleared tissue can only be imaged at a depth of <100 μm using two-photon microscope (Figures 2A,C), clearing of the spinal cord permitted imaging throughout its entire thickness (Figures 2B,D).

Using two-photon image analysis, we determined microglia/monocytes density in samples that previously underwent *ex vivo* diffusion MRI at acute (72 hours) and chronic stages (4 and 6 weeks) after complete SCI and compared to that of un-injured controls. Microglial/monocytes density was evaluated in CX3CR1^+/eGFP^ mice at distances of 0–500, 500–1,000, and 4,000–4,500 μm from the lesion site (Figure 3). The increase in microglia/monocytes density was particularly evident at acute compared to more chronic stages (Figure 3). In addition, as expected, areas adjacent to the lesion site displayed a greater increase in microglia/monocytes density than more distal regions (Figure 3). Quantitative analysis revealed significant increases in microglia/monocytes density at 72 hours and 4 weeks post-lesion compared to non-injured controls (Figure 3A). By 6 weeks post-injury, microglia/monocytes density decreased considerably, retuning to non-injured control level at 4 mm distal to the lesion site (Figure 3A). Overall, microglia/monocytes density was similarly elevated both rostral and caudal to the lesion site (Figures 3B,C).

Together, these data show (1) there is a greater increase in microglial/monocytes density contiguous to the lesion compared to more distal regions, (2) the highest microglial/monocytes density is observed at 72 hours post-injury, followed by a decrease at chronic stages after SCI, and (3) there is generally a similar increase in microglial/monocytes density after injury rostral and caudal to the lesion site.

### Changes in microglial/monocytes somata morphology are different rostral and caudal to the lesion site

**Figure 4:**
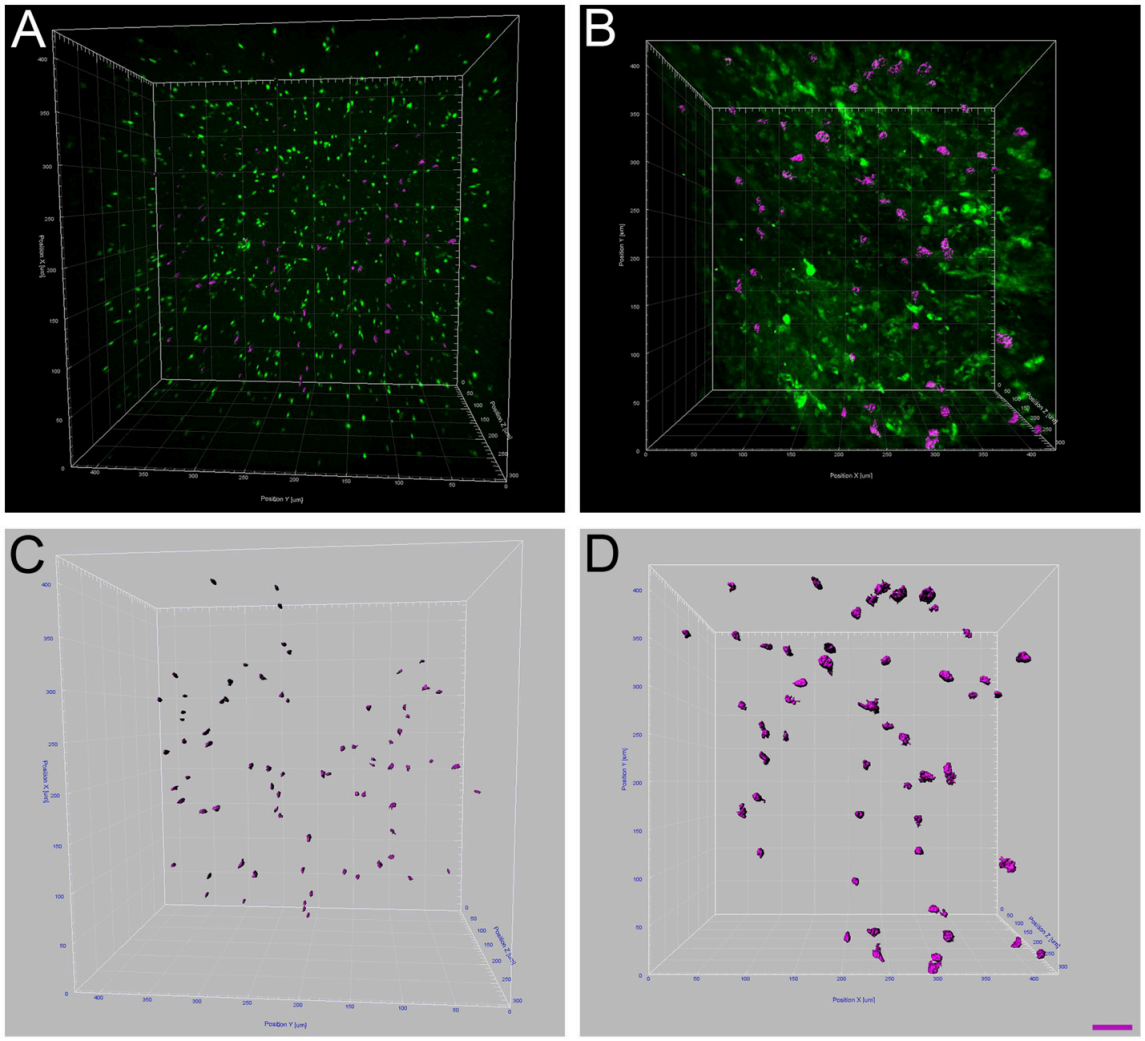
Increased microglia/monocytes activation after SCI. Two-photon micrographs displaying representative eGFP-positive microglia used for morphological analysis **(A,B)**. A sub-population of microglia/monocytes somata with no overlapping was selected (pink objects in **C**,**D**) for morphological analysis from both non-injured control **(A,C)** and injured animals **(B,D)**. 3D reconstruction and Imaris-based quantification of microglia/monocytes somata morphology in CX3CR1^+/eGFP^ mice **(C,D)**. Scale bars **(A–D)**: 50 μm.

**Figure 5:**
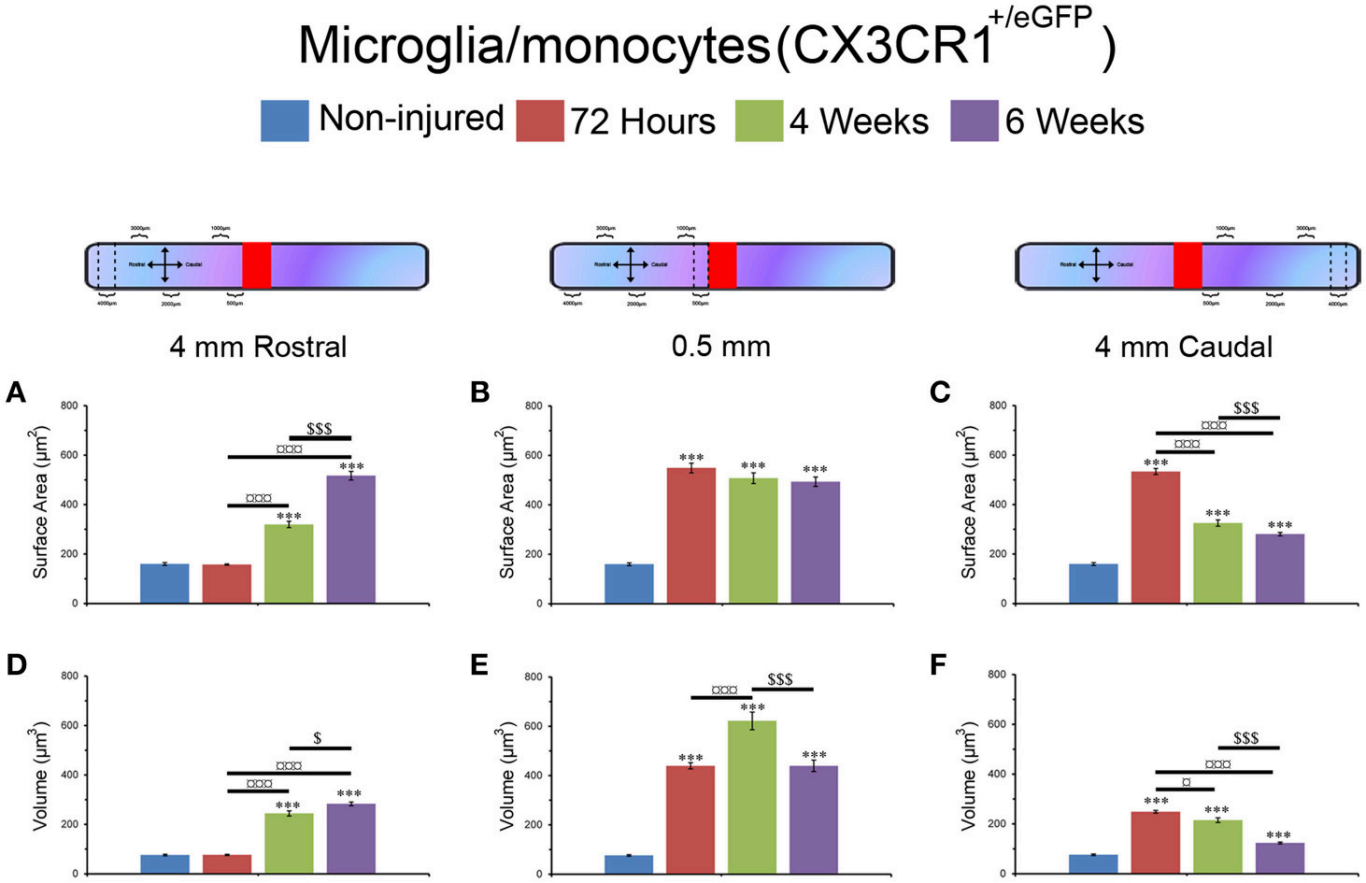
Morphological alterations of microglia/monocytes after SCI. Schematic drawing of longitudinal spinal cord after transection illustrating the lesion site (red rectangle) and the different zones (dashed lines) where microglia/monocytes morphologies were measured. Bar graphs indicating SCI-induced alterations in microglial/monocytes somata surface area **(A–C)** and volume **(D–F)** at different time-points after lesion. A pronounced increase in microglial/monocytes somata surface area and volume was evident at 500 μm rostral to the lesion epicenter as early as 72 hours post-lesion that continued until 6 weeks post-lesion **(B,E)**. At 4 mm rostral to the lesion site, microglia/monocytes displayed a time-dependent increase starting at 4 weeks **(A,D)**, whereas 4 mm caudal to the lesion there was a time-dependent decrease in microglia/monocytes somata surface area and volume starting at 72 hours **(C,F)**. ^***^P < 0.001 compared to un-injured control, ^*^P < 0.05, ^***^P < 0.001 compared to 72 hours post-lesion, ^$^*P* < 0.05 and ^$$$^*P* < 0.001 compared to 4 weeks post-lesion group, un-paired *t*-test.

Microglia/monocytes activation is characterized by a pronounced modification of their morphology. We thus quantified the surface area (μm^2^) and volume (μm^3^) of the somata of at least 190 individual cells per time-point. Only microglia/monocytes without overlapping somata with their neighboring cells were selected for morphological analysis (Figure 4, *pink objects*). Analyses were done at 500 μm rostral to the lesion site as well as 4 mm both rostral and caudal to the lesion epicenter (Figure 5). The increase in microglia/monocytes somata surface area and volume was particularly evident in the 500 μm segment rostral to the lesion than in more distant regions on both the rostral and caudal sides (Figure 5). Quantitative analysis of microglia/monocyte soma surface area and volume in proximity to the lesion site revealed a significant increase from 72 hours to 6 weeks post-injury (Figures 5B,E). Rostral to the lesion site, the size of microglia/monocytes somata displayed a time-dependent increase (Figures 5A,D), while caudal to the lesion site there was a continuous decrease in somata surface area and volume (Figures 5C,F). Thus, time course analysis of microglia/monocytes somata morphology revealed opposite responses rostral (Figures 5A,D) and caudal (Figures 5C,F) to the lesion site.

Together, these findings demonstrated that (1) microglia/monocytes display over a 5-fold increase in average somata size adjacent to the lesion site that remains elevated up to 6 weeks post-lesion, (2) microglia/monocytes rostral to the lesion site display a time-dependent increase in somata size, and (3) microglia/monocytes caudal to the lesion site display a time-dependent decrease in somata size. We thus highlight variation between segments located symmetrically rostral and caudal to the lesion site.

### Microglial/monocytes density coincides with lesion extension following complete SCI

We then investigated the potential link between injury-induced tissue re-organization and neuroinflammation in the injured spinal cord by examining putative correlation between *ex vivo* diffusion MRI and microglia/monocytes density. The increase in lesion extension, which was greatest at 72 hours post-injury amongst all the time points analyzed, coincided with the upsurge of microglia/monocytes density (Figures 1E, 3A and Supplementary Figure 2).

Lastly, we compared ADC values with the density and morphology of microglia/monocytes somata (Supplementary Figure 2). Rostral to the lesion site, the ADC at all time-points post-injury was similar to that of the non-injured control (Figure 1G). At the lesion site, ADC increased in a time-dependent manner (Figure 1G). All injured groups, except at 4 weeks post-injury (Table 1), displayed a higher ADC caudal to the lesion than the non-injured control. Unlike ADC, microglia/monocytes somata showed changes in surface area and volume both rostral and caudal to the epicenter (Figures 1G, 5 and Supplementary Figure 2). Thus, ADC does not seem to be correlated with microglia/monocytes somata morphology.

## Discussion

In the current study we performed a transection of the spinal cord in transgenic mice that express eGFP in microglia/monocytes (CX3CR1^+/eGFP^). We then carried out *ex vivo* diffusion MRI analyses of the spinal cord at acute (72 hours) and chronic (4 and 6 weeks) stages after the lesion, followed by tetrahydrofuran-based clearing and quantitative assessment of microglia/monocytes density and somata morphology using two-photon microscopy. Diffusion MRI offers better contrast than T2 weighted images allowing a more accurate identification of damaged tissues. Our findings suggest an early increase in the extension of the lesion that is significantly reduced by 4 weeks post-injury. Interestingly, the increase in lesion extension positively coincides with an increase in microglia/monocytes density. We also observe differences in microglia/monocytes somata morphology rostral and caudal to the lesion site that is compatible with cell debris and/or myelin clearance. This is the first study that combines *ex vivo* diffusion MRI with two-photon microscopy to document spatiotemporal changes in microglia/monocytes after severe SCI.

### Microglia/monocytes density after severe SCI

Despite the established presence of neuroinflammation in SCI pathophysiology, only a few studies have investigated post-injury microglia/monocytes density in mice, and findings are contradictory (Schnell et al., 1999; Kigerl et al., 2006; Stirling and Yong, 2008). A peak increase in microglia/monocytes density had been shown between 7 and 14 days post-injury (Schnell et al., 1999; Kigerl et al., 2006) but has also been reported as early as 2 days post-lesion (Stirling and Yong, 2008). In addition to the different techniques used to quantify microglia/monocyte density, differences in mouse strains may also account for these discrepancies (Basso et al., 2006; Lapointe et al., 2006). Using tissue clearing, Erturk and colleagues more recently reported a pronounced increase in microglia/monocytes density 10 days after spinal cord hemisection in mice (Erturk et al., 2012). Our quantitative analysis in severely injured spinal cords expands previous reports and provides a comprehensive analysis of microglia/monocytes density not only at acute and chronic stages after SCI but also at different distances from the lesion epicenter. Specifically, we observed that microglia/monocytes density is increased at 72 hours post-injury, then significantly reduced by 4 and 6 weeks with no major differences between the regions rostral and caudal to the lesion site. Although the pronounced increase in microglia/monocytes density at 72 hours post-injury may be attributed to infiltrating monocytes, CNS resident microglia can proliferate in the absence of peripheral monocytes (Elmore et al., 2014). Recent molecular study from our laboratory also confirmed that microglia are primarily involved in proliferation at 72 hours post-lesion (Noristani et al., 2017). In fact, resident microglia display greater proliferation compared to infiltrating monocytes after SCI (Greenhalgh and David, 2014). The increase in microglia/monocyte density at 72 hours occurred concomitantly with the greatest lesion extension observed by diffusion MRI. Modifications in diffusion MRI represent changes in the displacement of water molecules within different tissue compartments of the spinal cord. Thus, the positive association between microglia/monocytes density and lesion extension most likely corresponds to the early vasogenic edema resulting from plasma leakage and concomitant monocyte infiltration/microglia proliferation. Given that CX3CR1 is expressed both in infiltrating monocytes and resident microglia, in the current study, we cannot distinguish the exact proportion of these cells after SCI.

### SCI induces pronounced alterations in microglia/monocytes morphology

**Figure 6:**
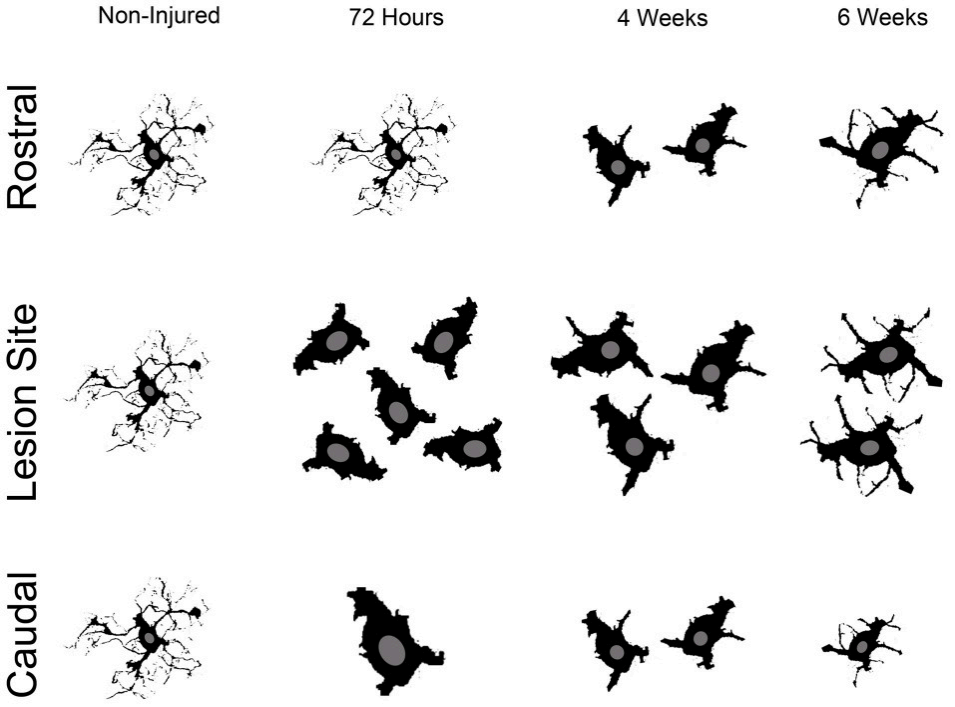
Schematic cartoon illustrating the overall microglial/monocytes response after SCI rostral and caudal to the lesion epicenter. Rostral to the lesion site, microglia/monocytes undergo gradual activation that continues up to 6 weeks post-lesion. Adjacent to the lesion site, there is a pronounced increase in microglia/monocytes density and reactivity. Subsequently, microglia/monocytes density is reduced adjacent to the lesion site; however, their increased reactivity is sustained at more chronic stages after injury. Caudal to the lesion site, pronounced microglia/monocytes reactivity is evident early after lesion and is then reduced at more chronic stages.

Although numerous studies have reported microglia/monocytes activation after injury (David and Kroner, 2011), no study to our knowledge has carried out quantitative analysis of injury-induced alteration of microglia/monocytes morphology. The most notable morphological alterations include a more than 3-fold increase in somata surface area and a more than 6-fold increase in somata volume that is most evident adjacent to the lesion site. The increase in somata volume induced by SCI is considerably higher than that reported in microglia following physiological stimulation, such as voluntary wheel running (Rodriguez et al., 2015). Interestingly, microglial/monocytes somata morphology distal to the lesion epicenter showed a clear difference in the regions rostral and caudal to the injury site (see Figure 6). Rostral to the lesion site, microglia/monocytes displayed a time-dependent increase in their reactivity that continued up to 6 weeks post-injury. In contrast, the greatest microglia/monocytes reactivity was observed caudal to the lesion site at 72 hours and decreased progressively between 4 and 6 weeks post-lesion. Measurements of ADC rostral and caudal to the lesion site also showed strong differences. Specifically, the ADC rostral to the lesion remained similar to that of un-injured controls throughout the 6 weeks following spinal cord trauma. In contrast, the ADC caudal to the lesion was significantly higher in all injured groups than the non-injured control. In the spinal cord, water diffuses in the white matter along the fibrous myelinated axons, which are orientated in a rostro-caudal direction. Diffusion MRI parameters, such as ADC are very sensitive to the changes in axonal integrity that occur during demyelination and inflammation (Stroman et al., 2014). Thus, an elevated ADC caudal to the lesion site might reflect demyelination. Indeed, even though limited and/or inadequate re-myelination may occur caudal to the lesion site after incomplete SCI (Alizadeh et al., 2015), this does not occur after a transection of the spinal cord. Microglia are predominantly involved in phagocytosis of damaged myelin sheaths and clearance of debris of axons caudal to the injury site that undergo Wallerian degeneration. The time-dependent decrease in microglia/monocytes reactivity caudal to the lesion site might thus reflect reduced myelin/debris clearance that is almost completed by 6 weeks post-injury. Rostral to the lesion site, the time-dependent augmentation in microglia/monocytes reactivity up to 6 weeks post-injury may correspond to a dying-back phenomenon and/or abortive regeneration. Indeed, while the distal segment of a cut axon undergoes Wallerian degeneration, the proximal segment dies back over a period of few days or weeks. At the tip of the axon rostral to the lesion site, “retraction balls” form and are physically disconnected from the axon. Moreover, the edge of the cut axon presents a structure that resembles a growth cone but is “dystrophic” (Steward, 2014), suggesting that there is a persistent regeneration attempt by the injured axon. However, since injured axons do not regrow to their targets, this regenerative process had been named “abortive regeneration” by Ramón y Cajal (1907). We thus hypothesize that the activated microglia/monocytes we observed rostral to the lesion site at 4 and 6 weeks post-lesion correspond to a delayed clearance of debris resulting from dying back and/or abortive regeneration. In addition, we cannot exclude the possibility that there may also be a greater infiltration of monocytes from the periphery caudal to the lesion site compared to rostral.

In this study we show that the greatest lesion extension observed following spinal cord transection, quantified using *ex vivo* diffusion MRI, occurs at 72 hours post-injury and coincides with changes in microglia/monocytes density but not with somata morphology. Using cleared spinal cords and two-photon imaging we also observed differences over time in microglia/monocytes somata morphology which, combined with diffusion MRI results, is compatible with time-dependent phagocytosis of debris by microglia/monocytes rostral and caudal to the lesion site.

## Conclusion

Our study confirms that the combination of *ex vivo* diffusion MRI and two-photon microscopy may be used effectively to follow structural tissue alterations and their putative relation to a given cell population after CNS lesion. However, no direct correlation was found between microglia/monocytes morphology and ADC that may results from the different resolution scales of the two methods. Analysis with the two-photon microscopy highlighted that differential microglia/monocytes reactivity caudal and rostral to the lesion may correspond to temporal differences in clearing processes and/or remaining of non-damaged axons. Our study points out the necessity of adjusting therapeutic strategies targeting microglia/monocytes after SCI to time post-injury and location of the lesion site.

## Author contributions

HN conceptualized the research, participated in the design of the study, performed all experiments, analyzed the data and contributed to the writing of the manuscript; HB participated in the design and analysis of the two-photon study; GS participated in MRI acquisition and analysis; PA participated in spinal cord clarification and in two-photon acquisition and analysis; RS participated in the design of the MRI coil; NL participated in SCI experiments; EA participated in MRI acquisition; NT participated in two-photon experiments; CG participated in MRI acquisition and analysis; CC participated in the design of the MRI coil and FP conceptualized the research, designed the project, participated in the analysis and interpretation of data, drafting the work and final approval. All authors read and approved the final manuscript.

### Conflict of interest statement

The authors declare that the research was conducted in the absence of any commercial or financial relationships that could be construed as a potential conflict of interest.

## Supplementary material

Supplementary Figure 1 *Ex vivo* MRI assessments following SCI. *Ex vivo* T2 weighted images (**B**, **D**, **F**, **H**, and **J**) and diffusion MRI (**A**, **C**, **E**, **G**, and **I**) from the same mouse spinal cord rostral **(A–D)** within **(E,F)** and caudal **(G–J)** to the lesion epicenter **(E–F)**. Panels **(C,D)** correspond to annotated images of **(A,B)**; **(I,J)** correspond to annotated images of **(G,H)**. Entire spinal cord (surrounded in green in **C**,**D** and **I**,**J**), damaged spinal cord tissues (surrounded in red in **E**, **F**, and **I**) and intact gray matter (surrounded in blue in **C**, **D** and **I**, **J**). Note that the superior contrast of DWI as compared to T2 weighted images allows better identification of damaged tissues **(G–J)**. Photograph of the custom-made ribbon solenoid coil used for *ex vivo* diffusion MRI acquisition **(F)**.

Supplementary Figure 2 Bar graph representations of Tables 2A–D.

Supplementary Video 1 A cleared spinal cord of an un-injured CX3CR1^+/eGFP^ mouse imaged with two-photon microscopy to assess microglia/monocytes density. Clearing of the spinal cord substantially improves the depth of two-photon imaging. For fully automated counting of microglia/monocytes, scans were converted into 3D objects. The animation was created using Imaris.

## Abbreviations

CNS: Central nervous system
MRI: magnetic resonance imaging
1H-MRI: proton MRI; diffusion weighted MRI (diffusion MRI)
eGFP: enhanced green fluorescent protein
PBS: phosphate base saline
PFA: paraformaldehyde
SCI: spinal cord injury
TR: repetition time
TE: Echo Time
NE: echo number
AVG: average
FOV: field of view
ADC: apparent diffusion coefficient.
