# Supplementary figures and images for: A Combination of Ex vivo Diffusion MRI and Multiphoton to Study Microglia/Monocytes Alterations after Spinal Cord Injury
Source: Front Aging Neurosci. 2017 Jul 19;9:230. doi: 10.3389/fnagi.2017.00230 (PMC5515855; doi:10.3389/fnagi.2017.00230)

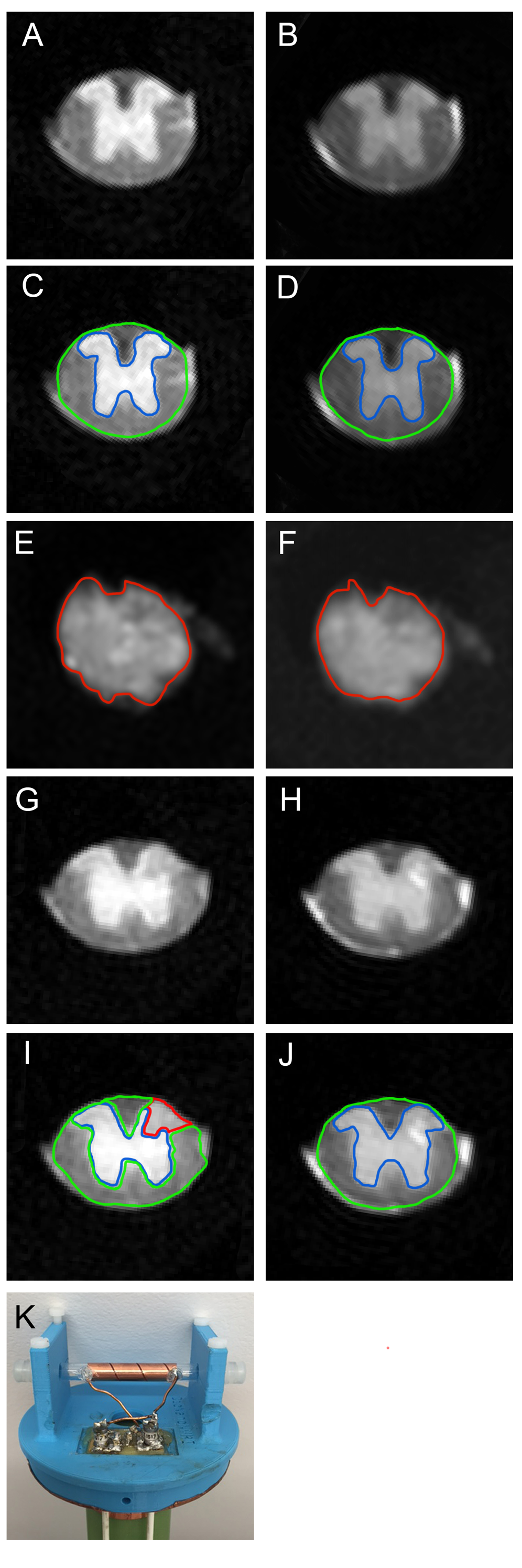

Supplement: Supplementary Figure 1 — Ex vivo MRI assessments following SCI. Ex vivo T2 weighted images (B, D, F, H, and J) and diffusion MRI (A, C, E, G, and I) from the same mouse spinal cord rostral (A–D) within (E,F) and caudal (G–J) to the lesion epicenter (E–F). Panels (C,D) correspond to annotated images of (A,B); (I,J) correspond to annotated images of (G,H). Entire spinal cord (surrounded in green in C,D and I,J), damaged spinal cord tissues (surrounded in red in E, F, and I) and intact gray matter (surrounded in blue in C, D and I, J). Note that the superior contrast of DWI as compared to T2 weighted images allows better identification of damaged tissues (G–J). Photograph of the custom-made ribbon solenoid coil used for ex vivo diffusion MRI acquisition (F). [file Image1.TIF]
