# Supplementary material for: A Combination of Ex vivo Diffusion MRI and Multiphoton to Study Microglia/Monocytes Alterations after Spinal Cord Injury
Source: Front Aging Neurosci. 2017 Jul 19;9:230. doi: 10.3389/fnagi.2017.00230 (PMC5515855; doi:10.3389/fnagi.2017.00230)

Supplementary illustration 2: comparison of modalities obtained by diffusion MRI and two-photon microscopy.
Rostral and caudal refer to the lesion site.

Table 2a. Apparent diffusion coefficient. Percentages at different time-points represent the variation as compared to the non-injured spinal cord.

**ADC**	**4mm rostral**	**1mm rostral**	**1mm caudal**	**4mm caudal**
**Non-injured**	100%		100%	
**72 hours**	+4%	+5.5%	+9%	+27.6%
**4 weeks**	+1.4%	+7.5%	+18.2%	+11.3%
**6 weeks**	+20%	+17.6%	+54.6%	+32.9%

Table 2b. Microglia/monocyte density. Percentages at different time points represent the variation as compared to the non-injured spinal cord.

**DENSITY**	**4mm rostral**	**1mm rostral**	**0.5mm rostral**	**0.5mm caudal**	**1mm caudal**	**4mm caudal**
**Non-injured**	100%					
**72 hours**	+12.8%	+331%	+294%	+322%	+352%	-10.3%
**4 weeks**	+37.6%	+50.3%	+77.6%	+75.7%	+68.9%	+32.8%
**6 weeks**	-2.4 %	+27%	+50.8%	+13%	+2%	-9.1 %

Table 2c. Lesion extension and volume.

	**Extension Total** (mm)	**Ext. rostral** (mm)		**Ext. caudal** (mm)	**Volume** Total (mm^3)	**Vol. rostral** (mm^3)	**Vol. caudal** (mm^3)
**72 hours**	4 ± 0.408	2		2	5.3 ± 0.89	2.5	2.8
**4 weeks**	2.7 ± 0.25	1.2		1.5	4.8 ± 0.77	2.3	2.5
**6 weeks**	3 ± 0	1.7		1.7	5.9 ±1.025	2.8	3.0

Table 2d. Microglia area, volume and sphericity. Percentages at different time points represent the variation as compared to the non-injured context.

	**Area**			**Volume**		
	Rostral 4mm	Rostral 0.5mm	Caudal 4mm	Rostral 4mm	Rostral 0.5mm	Caudal 4mm
**Non-injured**	100%			100%		
**72 hours (%)**	-1.6	+243	+234	+0.8	+476	+226
**4 weeks (%)**	+100	+218	+104	+220	+715	+182
**6 weeks (%)**	+223	+209	+76	+271	+476	+62

Additional Figure 2. Bar graph representations of table 2a-2d

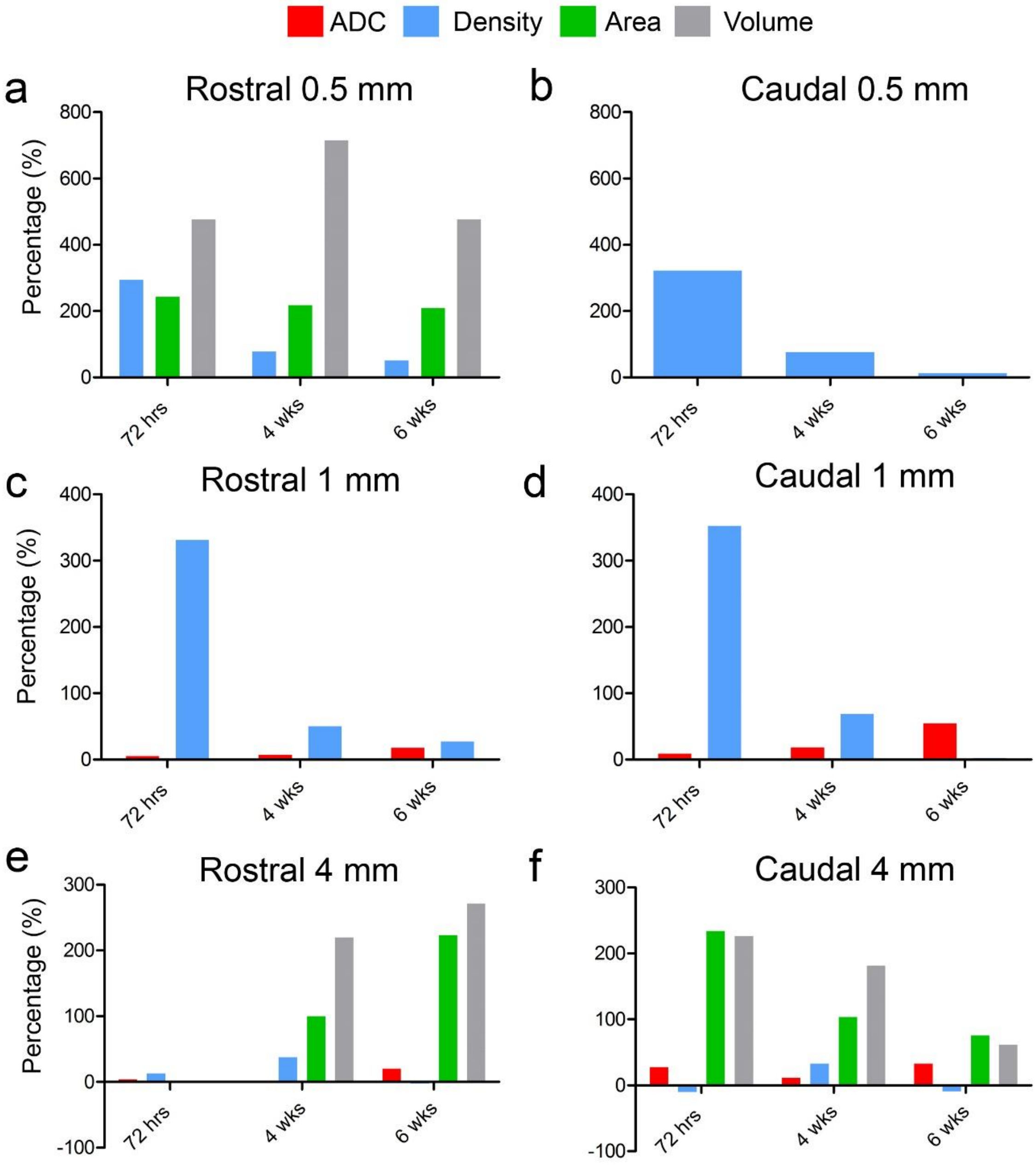

Supplement: Supplementary Figure 2 — Bar graph representations of Tables 2A–D. [file Image2.PDF]
